# The prognostic role of tumor size in stage T1 gastric cancer

**DOI:** 10.1186/s12957-022-02596-0

**Published:** 2022-04-27

**Authors:** Yan Chen, Yukun Jia, Zhan Peng, Guangye Wang

**Affiliations:** The People’s Hospital of Baoan Shenzhen, Shenzhen, China

**Keywords:** Gastric cancer, Tumor size, T-stage, Survival analysis

## Abstract

**Background:**

The purpose was to assess the contribution of tumor size to the prognosis of patients with gastric cancer.

**Methods:**

Patient data were sourced from the Surveillance, Epidemiology, and End Results program (SEER) database. Cox proportional risk regression was performed to determine the prognostic role of tumor size. Kaplan-Meier curves were conducted to calculate survival curves. Consistency index (c-index) and subject exercise curve (ROC) were utilized to assess the predictive ability of each factor on the prognosis of gastric cancer.

**Results:**

Tumor size is preferable to other widely accepted prognostic clinical features in forecasting the survival of patients with gastric cancer.

**Conclusions:**

The discriminatory ability of tumor size at T1 stage is superior to many other clinical prognostic factors.

## Introduction

Gastric cancer is one of the most common cancers, accounting for 5.6% of new cases worldwide, and its mortality rate is the second highest among cancer mortality rates [1]. prognostic factors for gastric cancer to help determine the best treatment strategy and predict patient survival. The American Joint Committee on Cancer (AJCC) tumor-node-metastasis (TNM) staging system is the most broadly used prognostic classification system in clinical practice. However, clinical practice has found that in patients with the same TNM stage, their prognosis also varies greatly. Therefore, the inclusion of additional independent prognostic factors should be considered to improve the tumor staging system and increase the accuracy of prognostic prediction.

Tumor size was described as the maximum diameter of a tumor that could be easily and objectively measured, and tumor size has been proven to be an important independent prognostic factor for various malignancies [2–4], and in the TNM staging system, the “T” stage for solid tumors, including breast, lung, and liver cancer, contains tumor size [5, 6]. However, the prognostic value of tumor size in gastric cancer has not been adequately recognized. Saito et al. showed that tumor size, an independent prognostic factor for gastric cancer, accurately predicted patient survival [7, 8]. However, the current AJCC 7th edition T-staging of gastric cancer includes only a vertical index of the depth of tumor infiltration [9], and the role of tumor size is ignored, and tumor size as an important prognostic indicator of gastric cancer, the prognostic role of tumor size may be different according to the depth of infiltration, but the prognostic role and predictive ability of T tumor size in different T stages are still unclear at present.

This study analyzed the prognostic role of tumor size by extracting data from the SEER database of gastric cancer patients. We also compared the prognostic value of tumor size with other prognostic factors in different T-stage gastric cancers to explore the impact of T-stage on the predictive ability and prognostic role of tumor size.

## Material and methods

### Patient data

The study data were obtained from the SEER database maintained by the National Cancer Institute (SEER*Stat 8.3.5.1). It is a public, free, and annually updated clinical records platform containing demographic and oncology information on cancer patients from 18 US registries. Using the SEER database, we identified a total of 112,151 cases of primary gastric cancer diagnosed between 1975 and 2016. Inclusion criteria included (A) pathological diagnosis of gastric cancer, (B) patients undergoing surgical treatment, and (C) gastric cancer as the only primary tumor. Patients with missing information on AJCC 7th edition TNM staging of gastric cancer, tumor size, and follow-up were excluded. Ultimately, a total of 5953 patients with gastric cancer were included in this study.

### Statistical analysis

Tumor size was analyzed as a continuous variable and described by the median and Inter-Quartile Range (IQR), the remaining factors were analyzed as categorical variables. Using the X-Tile software, continuous variables were converted to categorical variables [10]. The study endpoint is overall survival (OS), with OS representing the length of time from the date of diagnosis or the start of treatment. Univariate and multivariate Cox proportional risk regression analyses were performed to confirm the independent prognostic role of these factors. The discriminatory ability of tumor size and other factors was assessed by using c-index and ROC curves [11, 12]. Variables with higher c-index and area under curve (AUC) represent better discriminatory ability or prognostic accuracy. Survival curves were calculated by Kaplan-Meier analysis. Statistical analyses were performed using all using R software (4.0.3). Bilateral *P* < 0.05 was regarded as statistically significant.

## Results

### Patient characteristics

The detailed clinical characteristics of the included patients are presented in Table 1. Of these, 2464(41.4%) patients were younger than 65 years, 2567(43.1%) were female; the percentages of patients with grades I, II, III, and IV were 5.2%, 24.9%, 67.24%, and 2.7%; the percentages of patients with M0 and M1 were 89.3% and 10.7%; the percentages of patients with N0, N1, N2, and N3 were 39.7%, 18.2%,17.2%, and 24.8%, respectively; and the median tumor size (IQR) was 5.1(2.5–6.5) cm.

**Table 1:** Baseline characteristics of patients with different T-stage gastric cancer

	All (%)	T1 (%)	T2 (%)	T3 (%)	T4 (%)
Age, years					
≤65	2464 (41.4)	460 (33.5)	295 (40.5)	824 (42.7)	885 (46.0)
>65	3489 (58.6)	913 (66.5)	433 (59.5)	1106 (57.3)	1037 (53.9)
Sex					
Feman	2567 (43.1)	629 (45.8)	306 (42.0)	744 (38.5)	888 (46.2)
Man	3386 (56.9)	744 (54.2)	422 (58.0)	1186 (61.4)	1034 (53.8)
Grade					
I	309 (5.2)	201 (14.6)	31 (4.3)	49 (2.5)	28 (1.5)
II	1482 (24.9)	466 (33.9)	232 (31.9)	485 (25.1)	299 (15.5)
III	4000 (67.24)	679 (49.5)	444 (61.0)	1339 (69.3)	1538 (80.0)
IV	162 (2.7)	27 (2.0)	21 (2.9)	57 (3.0)	57 (3.0)
N stage					
N0	2366 (39.7)	1111 (80.9)	398 (54.7)	554 (28.7)	303 (15.8)
N1	1086 (18.2)	156 (11.4)	162 (22.3)	442 (22.9)	326 (17.0)
N2	1023 (17.2)	77 (5.6)	101 (13.9)	427 (22.1)	418 (21.7)
N3	1478 (24.8)	29 (2.1)	67 (9.2)	507 (26.3)	875 (45.5)
M stage					
M0	5315 (89.3)	1347 (98.1)	705 (96.8)	1749 (90.6)	1514 (78.7)
M1	638 (10.7)	26 (1.9)	23 (3.2)	181 (9.4)	408 (21.2)
Race					
Black	973 (16.3	218(15.9)	118 (16.2)	353 (18.3)	284 (14.8)
Other	1610 (27.0	464 (33.8)	201 (27.6)	460 (23.8)	485 (25.2)
White	3370 (56.6	691 (50.3)	409 (56.2)	1117 (57.8)	11,537 (60.0)
Tumor size (cm) Median (IQR)	5.1 (2.5–6.5)	2.4 (1.0–3.1)	3.8 (2.2–5.0)	5.6 (3.5–7.0)	6.9 (4.0–8.5)

*IQR* inter-quartile range

### Prognostic value of tumor size at different T-stages

**Fig. 1:**
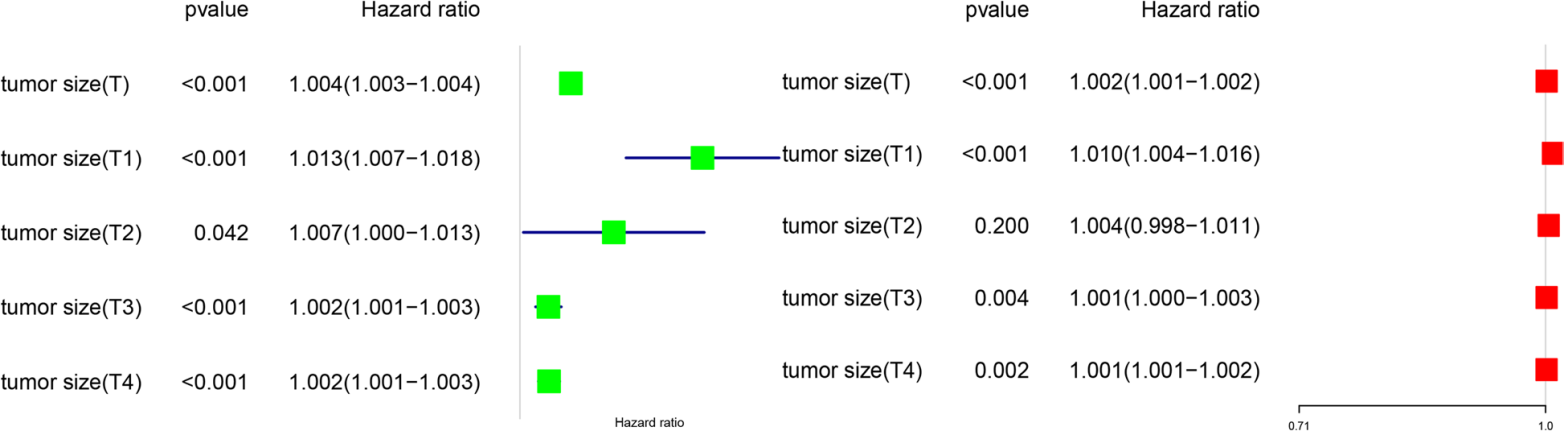
Effect of T-stage on the hazard ratio of tumor size to predict survival in gastric cancer. **A** Univariate. **B** Multifactorial

**Fig. 2:**
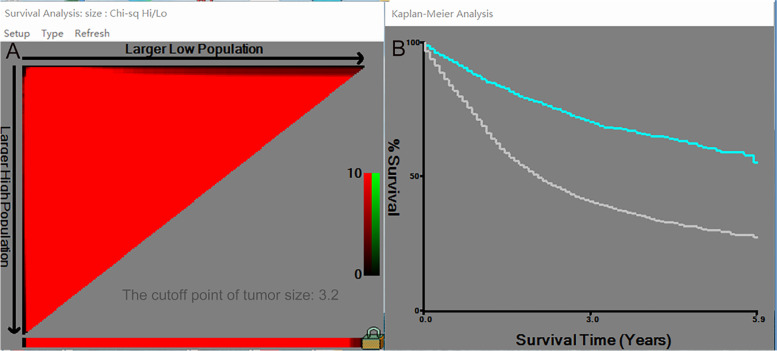
Tumor size cut-off points generated by X-Tile software. **A** The cut-off point generated by X-Tile software is 3.2cm. **B** The survival curve made according to the cut-off point

**Fig. 3:**
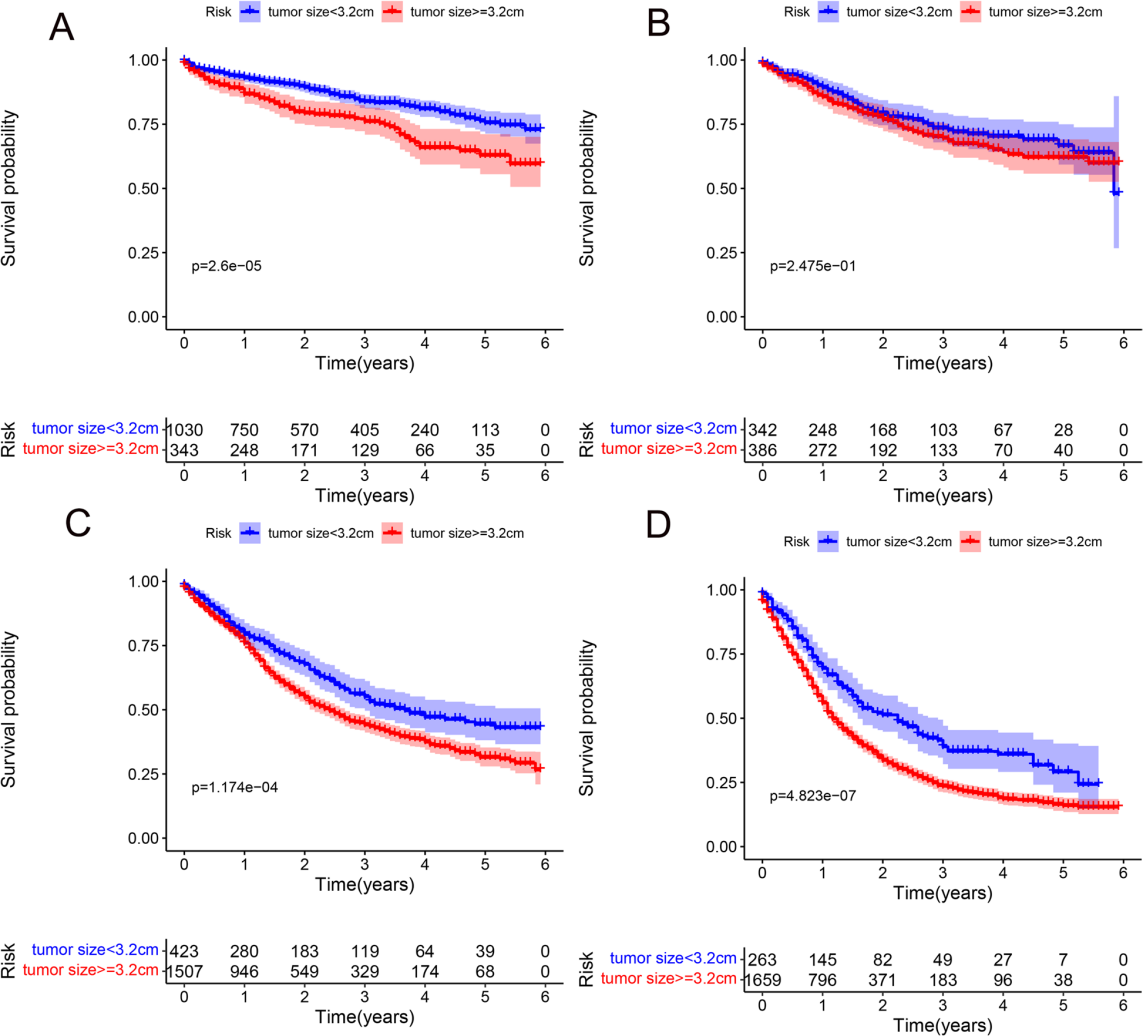
Kaplan-Meier survival curves comparing survival times at different T-stages for patients with gastric cancer less than 3.2 cm and greater than or equal to 3.2 cm: **a** T1 stage, **b** T2 stage, **c** T3 stage, and **d** T4 stage

The prognostic role of tumor size was assessed by univariate and multivariate Cox proportional risk regression analysis (Fig. 1). The results found that T-stage can influence the independent prognostic value of tumor size. Tumor size was an independent prognostic factor for T1, T3, and T4 gastric cancer, and HR was highest in T1 stage, but the HR was close to 1 in T3 and T4, indicating that the prognostic significance of tumor size was weaker in T3 and T4 than in T1. We then divided the cut-off values for tumor size according to the X-Tile (Fig. 2) and plotted survival curves at each T-stage according to tumor size to more visually demonstrate the differences in survival outcomes (Fig. 3). The results presented a significant difference in survival between the two groups (tumor size <3.2 cm, tumor size ≥3.2 cm) in stage T1, T3, and T4. However, there was no significant difference in survival outcomes in stage T2.

### Discriminatory ability of tumor size in different T-stages

The prognostic discriminatory ability of tumor size and other factors were compared by C-index and AUC (Table 2). Subgroup analysis according to different T stages revealed that tumor size C-index was higher at T1 stage (0.636) than age (0.548), sex (0.507), grade (0.550), M (0.571), and race (0.513), and tumor size was a valuable prognostic factor, superior to other widely used prognostic factors at T1 stage. However, in other T-stages, the predictive power of tumor size was not significant.

**Table 2:** Discriminatory ability of each factor to predict survival in gastric cancer

	ALL		T1		T2		T3		T4	
	C	ROC	C	ROC	C	ROC	C	ROC	C	ROC
Tumor size	0.636	0.623	0.584	0.596	0.545	0.596	0.549	0.555	0.549	0.554
Age	0.548	0.630	0.585	0.639	0.583	0.680	0.565	0.651	0.565	0.631
Sex	0.507	0.530	0.543	0.504	0.509	0.500	0.503	0.500	0.509	0.529
Race	0.513	0.500	0.510	0.500	0.508	0.500	0.505	0.500	0.511	0.500
N	0.657	0.576	0.550	0.531	0.596	0.635	0.595	0.532	0.574	0.520
M	0.571	0.591	0.534	0.541	0.525	0.511	0.551	0.591	0.562	0.578
Grade	0.550	0.522	0.511	0.500	0.508	0.500	0.520	0.515	0.518	0.515

*ROC* receiver operating characteristic curve, *C* concordance index

### Construction of tumor size-based nomogram in T1 gastric cancer

**Fig. 4:**
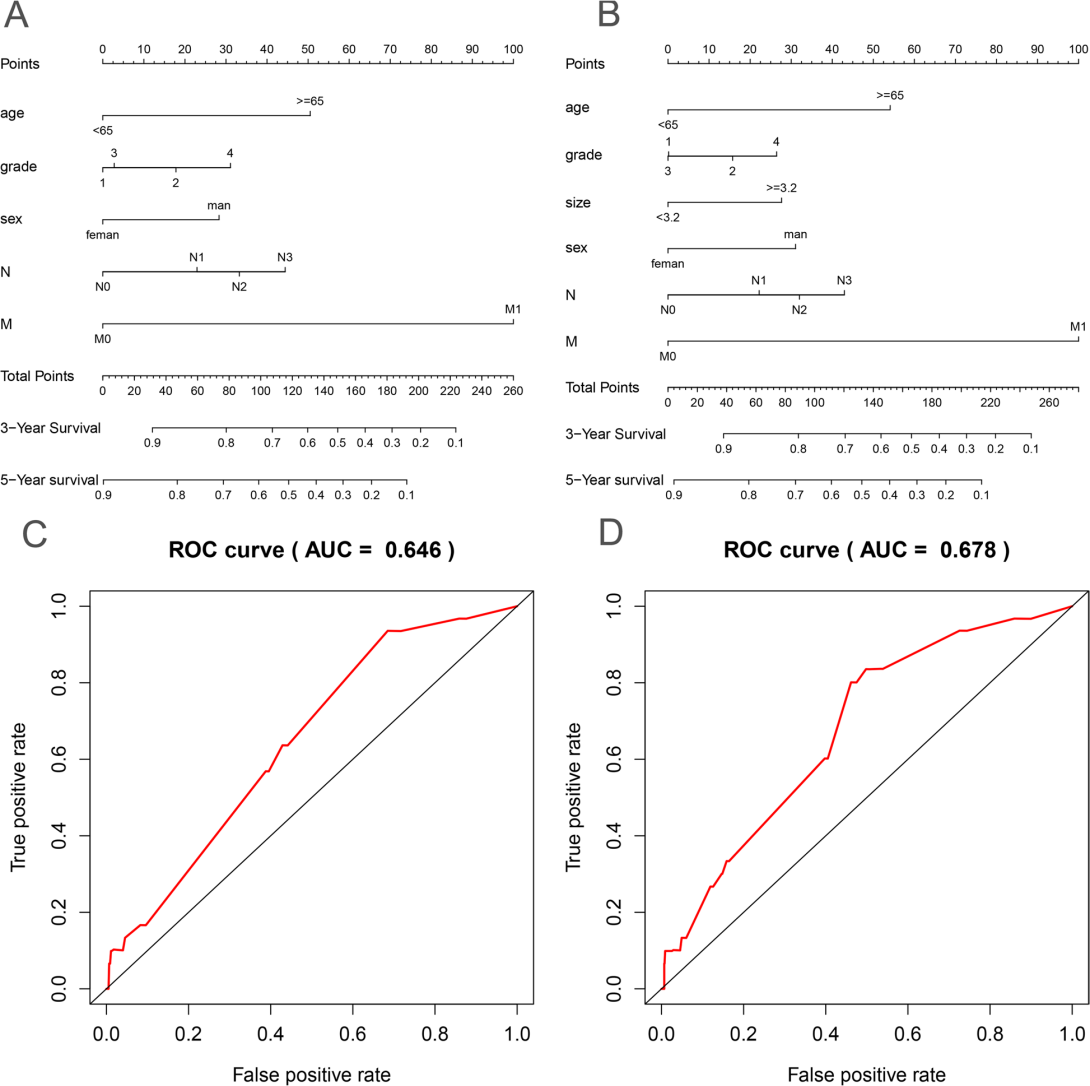
Construction of tumor size-based nomogram in T1 gastric cancer. **A** Nomogram without tumor size. **B** Nomogram based on tumor size. **C** Time-dependent ROC of nomogram without tumor size. **D** Time-dependent ROC of nomogram with tumor size

To further explore the clinical application of tumor size in the TNM system, we developed a nomogram based on multivariate Cox analysis to explore the significance of increasing tumor size in early-stage T gastric cancer. N stage, M stage, age, sex, and Grade stage were included in the nomogram (Fig. 4A), while tumor size, N stage, M stage, age, sex, and Grade stage were included in the nomogram (Fig. 4B). Time-dependent ROC showed that nomograms that included tumor size were significantly more accurate in predicting OS compared to models without tumor size (AUC at 5 years: 0.678 versus 0.646).

## Discussion

From the current researches on gastric cancer, it is known that a variety of prognostic factors can affect the survival rate of gastric cancer patients: age, tumor stage, tumor size, radiotherapy, chemotherapy, surgical resection, venous invasion, Helicobacter pylori eradication, preoperative systemic immune inflammation index, tumor deposits and some miRNA (miR-23b, ETS1, and TCF4) [13–18], among these factors, the prognostic value of tumor size is often overlooked, and furthermore, consensus on the optimal cut-off point for tumor size is difficult. In the TNM staging of AJCC, tumor size is included in the T-staging of many tumors. However, in the T-stage of gastric cancer, only an indicator of the depth of tumor infiltration is included. Usually, the direction of primary gastric cancer growth includes along the stomach wall and perpendicular to the stomach wall. The former forms the tumor size, while the latter is the depth of infiltration. The integration of tumor size into the T-staging system of gastric cancer is controversial, although many previous studies have demonstrated that tumor size is a non-negligible prognostic factor for gastric cancer and that including tumor size in the staging system improves the prognostic prediction of gastric cancer [19, 20]. However, they did not consider the effect of depth of infiltration on the prognostic significance of tumor size because many variables are interrelated and the effect of tumor size on prognosis can be accurately assessed only if the depth of tumor infiltration is clearly defined. In other words, the optimal threshold for tumor size is different at different depths of infiltration (T-stage). The results of this study also demonstrated the different prognostic values of tumor size in different T-stages. The results of K-M analysis showed similar results, with a significant difference in survival between patients with tumor size less than 3.2 cm and greater than 3.2 cm at stage T1, in contrast, at the T2 stage, the two groups showed no statistically significant differences between them. This means that the effect of tumor infiltration depth cannot be ignored while considering the prognostic value of tumor size.

This paper investigated the prognostic significance of tumor size at different T-stages. The results indicated that consistent with previous findings, tumor size was an important independent prognostic factor for gastric cancer [7, 8, 21–23]. However, these studies did not elucidate the prognostic value and discriminatory ability of tumor size in different T-stages. An increase in T staging may negatively impact the value of tumor size on prognosis. In patients with T1 stage, larger tumor size was related with a poorer prognosis. In addition, in patients with stage T1 gastric cancer, the predictive power of tumor size was superior to many other widely used prognostic factors. However, tumor size is not an independent prognostic factor for stage T2 gastric cancer, and the discriminatory power to predict OS was significantly weaker in patients with more advanced T staging.

There are two possible reasons for the negative effect of T staging on the prognostic role of tumor size. First, the calculation of tumor size may be inaccurate when the tumor invades below the submucosa. On the other hand, when the tumor is confined to the mucosa and submucosa (stage T1), the predominant tumor growth pattern is horizontal, and as the tumor infiltrates the intrinsic muscular layer, vertical growth is the predominant growth pattern. Therefore, in stage T3–4 cancers, tumor size has a lower prognostic value in T3 and T4 gastric cancers than in stage T1.

The most interesting finding of this study is that tumor size as a predictor for patients with stage T1 gastric cancer outperformed many widely used clinical prognostic factors in terms of predictive power. Early gastric cancer was defined as a cancer with an infiltration depth limited to the mucosal or submucosal layer (stage T1) [24], and whether the lymph nodes metastasize or not is not relevant to the classification. The most serious prognostic factor for early gastric cancer is currently considered to be lymph node metastasis [24], while some studies have shown that the metastasis of lymph nodes is related to the size of the tumor [25, 26], and tumor size over 3.0 cm is significantly correlated with lymph node metastasis [27]. The frequency of lymph node metastasis in early gastric cancer is 2–3% (mucosal carcinoma) and 15–20% (submucosal carcinoma) [25–33]. Liang et al. showed that tumor size was also an independent prognostic factor after surgery in patients with gastric cancer without lymph node metastasis, and that tumor size improved the accuracy of prognostic prediction in patients [34]. This provides strong evidence to support that tumor size can be applied to future revisions of the AJCC TNM staging system for stage T1 gastric cancer.

This study has several limitations: first, the inherent limitations of retrospective studies. Second, no external data validation was performed. Third, due to the limitations of the final number of patients included, only patients were divided into two groups, and no further studies on the optimal cut-off values were performed. Fourth, the study did not consider patients with metastatic gastric cancer, which may have affected the results. Despite these limitations, as far as we know, the present study is the first to assess the impact of T staging on the prognostic and predictive value of tumor size in gastric cancer. Importantly, we found the prognostic value of tumor size in patients with stage T1 gastric cancer.

## Conclusions

Tumor size has a significant influence on the prognosis of stage T1 gastric cancer. In addition, the discriminatory ability of tumor size at the T1 stage is superior to many other clinical prognostic factors, the impact of tumor size on prognosis should be taken into consideration when evaluating the prognosis of gastric cancer patients. When considering the inclusion of tumor size in AJCCT staging, one needs to consider that the prognostic role of tumor size is different at different T-stages.

## Data Availability

Publicly available datasets were analyzed in this study. This data can be found here: Surveillance, Epidemiology, and End Results (SEER) database (https://seer.cancer.gov/).

## Abbreviations

AJCC: American Joint Committee on Cancer
TNM: Tumor-node-metastasis
c-index: Concordance index
ROC: Receiver operating characteristic curve
SEER: Surveillance, Epidemiology, and End Results program
IQR: Inter-quartile range
OS: Overall survival
HR: Hazard ratio
K-M: Kaplan–Meier
AUC: Area under curve
